# CCL20 Secretion from the Nucleus Pulposus Improves the Recruitment of CCR6-Expressing Th17 Cells to Degenerated IVD Tissues

**DOI:** 10.1371/journal.pone.0066286

**Published:** 2013-06-18

**Authors:** Wen Zhang, Lin Nie, Yan Wang, Xu-ping Wang, Hua Zhao, Samina Dongol, Sailendra Maharjan, Lei Cheng

**Affiliations:** 1 Department of Orthopedics, Qilu Hospital, Shandong University, Jinan, Shandong, China; 2 Department of Pathology, Qilu Hospital, Shandong University, Jinan, Shandong, China; 3 The Key Laboratory of Cardiovascular Remodeling and Function Research, Chinese Ministry of Education and Chinese Ministry of Public Health, Shandong University, Qilu Hospital, Jinan, Shandong, China; 4 Department of Obstetrics and Gynecology, Qilu Hospital, Shandong University, Jinan, Shandong, China; University Medical Center Freiburg, Germany

## Abstract

**Background:**

Studies elucidated that Th17 cells are important contributors to the pathogenesis of many immune-mediated diseases, and IL-17A is present in pathologic intervertebral disc (IVD) tissues. However, the mechanisms, how these cells traffic into the degenerate discs are not clear.

**Materials and Methods:**

The samples collected from 53 patients had been divided into 3 groups: Group P (annulus fibrosus was intact), Group E (annulus fibrosus was reptured) and normal control. Immunohistochemistry was used to detect the expression of CCL20, CCR6, IL-17A, TNF–α and CD4 in IVD tissues. Moreover, nucleus pulposus (NP) cells had been cultured in the presence and absence of Th17 associated cytokines. The supernatants were detected for CCL20 concentrations by ELISA, and the NP cells for the expression of CCL20 mRNA. Additionally, peripheral blood (PB) samples had undergone detection for the expression of CCR6 mRNA and the proportion of IL-17-producing cells, including the surface expression of CCR6.

**Results:**

Immunohistochemistry revealed that CCL20 and TNF-α were expressed in degenerated NP cells. Double-labeled immunofluorescence elaborated, IL-17-producing cells (CD4^+^IL-17A^+^ and CD4^+^CCR6^+^) appeared in the Group E samples, but no traces or expression in Group P and normal control. IL-17A and TNF-α, alone or combined, could enhance CCL20 secretion in a dose-dependent manner, which was obtained through RT-PCR results. There was a notable difference of CCR6 mRNA expression between patients and normal controls. In comparison to controls, flow cytometry data indicated that the proportion of IL-17-producing cells and the CCR6 expression in PB were significantly increased.

**Conclusion:**

Our results provide a potential explanation for involvement of the CCL20-CCR6 system in the trafficking of IL-17-producing cells to degenerated IVD tissues. Additionally, our results explain the contribution of Th17 associated cytokines to the development of degenerated discs via the up-regulation of CCL20 secretion from NP cells, which forms a positive chemotactic feedback loop.

## Introduction

Disc herniation is currently well established as an immune-mediated inflammatory process. The auto-immune theory of intervertebral disc (IVD) herniation, which is based on the particular anatomical structure, was first suggested by Naylar [1]. According to immunologists and Burnet’s clonal selection theory [2], the nucleus pulposus (NP), the largest avascular tissue *in vivo*, exists in an immuno-privileged state and, once prominently exposed to the immune system, it can not be recognized by our immune system. Instead, the NP elicits an immune response that stimulates auto-immunity and subsequently leads to chronic inflammation and sustained lumbocrural pain.

Previously, it was commonly thought that activated CD4^+^T cells differentiated into 2 subsets, Th1 and Th2. According to Mossman and Coffman, each subset possessed mutual functions and forms of cytokine secretion [3]. This classification method was upheld until 2005, when the IL-17A-producing T-cell subset (Th17) was discribed [4], [5]. Th17 cells are characterized by their production of IL-17A, which provokes a wide range of inflammatory reactions, but they also secrete many other inflammatory cytokines, such as IL-22, TNF-α (a well-known mediator of inflammatory disease) and IL-26 [6], [7], [8], [9]. Recently, numerous studies in human and mice have suggested that Th17 cells play a leading role in the pathogenesis of many different immune-mediated diseases, including rheumatoid arthritis [10], [11], psoriasis [12], [13], asthma [14], [15] and inflammatory bowel disease [16], [17]. The cells from those lesions, including synoviocytes, keratinocytes, bronchial epithelial cells and colon epithelial cells, can produce abundant amounts of CCL20, a specific ligand for CCR6. CCR6 is specifically expressed on the surfaces of Th17 cells, and is associated with Th17 infiltration [18], [19], [20].

Taken together, these research achievements suggest that the data might implicate a similar process for intervertebral disc degeneration. The specialized anatomical structure and immune-mediated inflammatory process indicate that Th17 cells might mediate the inflammation associated with intervertebral disc degeneration. Of particular interest is the recent report by Shamji [22], in which the Th17 associated cytokine IL-17A was observed in >70% of pathologic IVD tissues; this finding provided a marked contrast to the low levels of IL-17A observed in control tissues obtained from autopsies. Gabr [23] reported that the addition of IL-17A, IFN-γand TNF-α, alone or in recombination, to cultured IVD cells promoted the production of inflammatory mediators, such as NOx, PEG2 and IL-6. These results suggested that IL-17A might be an essential regulator of multilevel inflammatory responses, and that Th17 lymphocytes might play an important role in the pathology of IVD disease. However, whether IL-17-producing cells are present in the degenerated IVD lesions and the mechanisms behind the trafficking of these cells into the degenerated discs are currently unknown.

In the present study, we aimed to investigate the role of Th17 cells in the pathogenesis of IVD disease. First, we determined whether NP cells could secrete CCL20 and whether the expression of Th17 associated cytokines (IL-17A and TNF-α) was related to CCL20 secretion. Next, we evaluated the expression of TNF-α, CD4 and CCR6 by immunohistochemistry, and employed double immunofluorescence staining to confirm the presence or absence of infiltrating of IL-17-producing cells. Additionally, we used RT-PCR to detect the CCL20 mRNA levels in cultured NP cells and the CCR6 mRNA levels in the peripheral blood mononuclear cells (PBMCs) isolated from the pathologic IVD patients. Finally, we determined the proportion of IL-17-producing cells and the surface expression of CCR6 on those cells by flow cytometry.

## Materials and Methods

### Ethics Statement

Patient Enrollment took place between April 2012 and July 2012 at Qilu Hospital, Shandong University, China. Our research was approved by the Medical Ethical Committee of Qilu Hospital, Shandong University. A written informed consent document was obtained from each participant.

### Patients and Controls

Human pathological specimens, which were obtained from patients diagnosed with disc degeneration who were undergoing discectomy procedures, were mainly used in 2 crucial components of our research: immunohistochemistry (IHC) and cell culture. For IHC analyses, disc tissues were collected during surgery from 25 female patients (age range: 20–57 years, mean: 40.6±11.84 years) and 25 male patients (age range: 18–61 years, mean: 42.26±14.58 years) who have received diagnoses of disc degeneration at Qilu Hospital of Shandong Province, China. According to the surgical and imaging findings, we divided the specimens into two groups: the protrusion group, in which the annulus fibrosus (AF) was intact (Group P, n = 20) and the extrusion group, in which the AF was ruptured (Group E, n = 30). Before the surgeries, peripheral blood (PB) samples from the patients, designated as the patient group, and PB samples from 10 healthy controls, designated as the control group were collected. CCR6 mRNA expression was evaluated in the blood samples. Additionally, 3 disc tissue samples (2 male and 1 female) diagnosed with scoliosis were designated as the normal controls (age range: 16–31 years, mean: 24.33±7.64 years) for immunohistochemical staining. For the cell culture experiments, disc tissues were collected from 8 patients (5 male and 3 female, age range: 18–46 years, mean: 29.5±9.96 years); some of these patients underwent two level discectomies, including a posterior lumbar interbody fusion (PLIF) or microendoscopic discectomy (MED), while none of the patients underwent multilevel lumbar interbody fusion discectomies. Granular tissues were not present, and the NP could be clearly defined from the AF. For flow cytometry experiments, we collected PB samples from another 20 patients (age range: 38–61 years, mean: 51.63±8.21 years), which were designated as the patient group, and also collected PB samples from 15 healthy controls (age range: 21–42 years, mean: 31.47±6.42 years), which were designated as the control group. The proportion of IL-17-producing cells and the surface expression of CCR6 on those cells were evaluated in the blood samples. The criteria for the patient selection were as follows: typical osphyalgia or sciatica; positivity for Lasegue’s sign or the Bragard test; a reduction in the thickness of the degenerated disc on a spinal X-ray, a reduction or disappearance of the physiologic spinal curvature, a reduction in the signal of the involved NP on a T2-weighted MRI analysis, and no signs of calcification on the CT; no presence of tuberculosis, rheumatoid arthritis or other immunological disorders and no recent history of glucocorticoid use. The determination of disc degeneration grade was made according to the Pfirrmann grading system, which is based on the MR signal intensity, the disc structure, the distinction between the nucleus and annulus, and the disc height [24]; these criteria are shown in Table 1. The experimental protocol was approved prospectively by the human subjects institutional review board of Shandong University, and all patients signed an informed consent waiver for the use of the IVD tissues and blood samples.

**Table 1:** Summary of characteristics in the study population.

Characteristics		Group P	Group E	Normal controlof IVDs	Normal control of PB for RT-PCR	Normal control of PB for Flow cytometry	Patient group of PB for Flow cytometry	Cell culture
No. of patients		20	30	3	10	15	20	8
Age(years)	Mean	38.6±12.16	47±13.62	24.33±7.64	34.45±5.80	31.47±6.42	51.63±8.21	29.5±9.96
	Range	20–60	18–61	16–31	26–44	21–42	38–61	18–46
Male/female		11/9	14/16	2/1	5/5	9/6	11/9	5/3
Average degeneration grade		III	IV	II	I	I	III	III

### Nucleus Pulposus Cell Isolation and Culture

The discs that were collecteded from the PLIF/MED surgeries were transported immediately to the laboratory at 0°C in 50 ml sterile centrifugal tubes containing 30 ml of sterile DMEM/F12 culture medium with 10% fetal calf serum (FCS) (Gibco; Invitrogen corporation, USA), 1% penicillin-streptomycin (PS: 100 µg/ml each of penicillin and streptomycin). The tissues were rinsed three times with sterile Phosphate Buffer Solution (PBS) with 1% PS to remove any residual blood. Next, the NP tissues were carefully separated from the AF on the basis of their macroscopic morphology and minced into small fragments of about 1 mm^3^. The NP cells were isolated by a trypsin and Type II collagen enzyme digestion (Sigma-Aldrich, Trading Co., Ltd, China). The isolated cells were seeded as a monolayer primary culture at a density of approximately 25,000 cells/cm^2^ in DMEM/F12 (Gibco, Invitrogen corporation, USA) culture media with 10% FCS, 1% PS, 0.05% Fungizone, and 50 mg/ml of ascorbate under standard incubation conditions (37°C, 5% CO_2_, 95% air, bicarbonate buffering to maintain pH 7.2) for 3 weeks. The culture media was replaced twice per week, but the primary cells were allowed to adhere before the first change of the culture media [25]–[27]. When the cells were approximately 80%–90% confluent, the NP cells were dissociated with 5% trypsin (Sigma-Aldrich) plus 1% ethylenediaminetetraacetic acid (EDTA), and were subcultured at a ratio of 1∶2. Th17 associated cytokine stimulation experiments were performed on NP cells at passages 2 or 3; the cells were approximately 70%–80% confluent NP cells in 24-well plates and were maintained under the above-described conditions. The NP cells were cultured either alone or with one of the following cytokines: IL-17A at 1 ng/ml, 10 ng/ml, 100 ng/ml or 1000 ng/ml (PeproTech, USA) and TNF-α at 0.1 ng/ml, 1 ng/ml, 10 ng/ml, or 100 ng/ml (PeproTech, USA). At the appointed time intervals after receiving cytokine stimulation, the supernatants and NP cells were harvested for CCL20 protein detection and mRNA quantification, respectively.

### Isolation of the Peripheral Blood Mononuclear Cells (PBMCs)

Peripheral blood samples from the above-mentioned patients were centrifuged at 2000 rpm for 15 min, and the cells were mixed with equal volumes of normal saline solution. Equal volume of lymphocyte separation medium (Corning Cellgro, USA) were added to the mixtures and the cell suspension were centrifuged at 1500 rpm for 10 min. We collected the intermediate buffy coat from each sample, mixed the cells with a quadruple volume of normal saline solution and centrifuged the suspensions at 1500 rpm for 10 min. Finally, we washed the cells with an appropriate volume of saline and collected the sedimented cells, which were subsequently added to 1-ml Trizol per sample (Invitrogen, USA) and stored at –80°C for RT-PCR analysis.

### Immunohistochemistry (IHC) and Double Immunofluorescence Staining

We collected 50 IVD tissues (Group P, n = 20 and Group E, n = 30) for immunohistochemistry. Three normal discs from scoliosis patients were allocated to the control group. All of the disc samples were paraffin embedded and used for IHC and double immunofluorescence staining. The discs that were harvested from the PLIF surgeries were immediately rinsed three times with sterile normal saline (NS) to remove all residual blood. The NP tissues were carefully separated from the AF and were formalin-fixed and paraffin-embedded. The paraffin sections were warmed in an oven for 1 hour, de-paraffinized with xylol and rehydrated in a descending gradient ethanol series. Endogenous peroxidases in the sections were blocked with 3% hydrogen peroxide, and antigen retrieval was performed by microwaving the sections in EDTA (1∶50, ZSGB-Bio, China) at 95°C for 15 min. After blocking the nonspecific proteins with 20% goat serum (ZSGB-Bio, China), goat anti-human CCL20 (5 µg/ml, R&D Systems, Inc., USA) and rabbit anti-human TNF-α (Abcam Inc., USA) primary antibodies were added to the sections, which were incubated overnight at 4°C. Appropriate isotype matched goat or rabbit antibodies at the same concentrations as the primary antibodies were used as isotype controls (goat IgG, R&D Systems, Inc., USA; rabbit IgG, Abcam Inc., USA). Subsequently, the sections were treated with secondary antibodies (Abcam Inc., USA) and incubated at 37°C for 30 min, followed by the application of a 0,0-dimethyl-0-2,2-dichloroethylene phosphate tertiary linker for 20 min at 37°C. Staining was detected with 3, 3′-diaminobenzidine (DAB, Abcam Inc., USA), followed by a distilled H_2_O rinse and dehydration in an ascending gradient ethanol series and xylol. The slides were cover slipped with a synthetic xylol-based mounting medium, and the patterns of immunoreactivity were visualized on a IX71-SIF type microscope (Olympus, Japan). Then we selected 3 random microscopic fields per-slide and used the ImagePro Plus 5.0 software (Media Cybernetics, MD, USA) for digital photographs analysis. In short, the positive site which was stained to brown was measured in pixels using the software and we got 3 measurements: area, mean density (MD) and integrated optical density (IOD). All digital photographs were taken and measured in the same parameter setting to insure these data were comparable. The mean density (MD) was defined as the expression intensity of those cytokines.

For double immunofluorescence staining, mouse anti-human CCR6 (6.25 µg/ml, R&D Systems, Inc., USA), rabbit anti-human CD4 (1∶200, Abcam Inc., USA), rabbit anti-human IL-17A (5 µg/ml, R&D Systems, Inc., USA) and mouse anti-human CD4 (1∶200, Abcam Inc., USA) primary antibodies were all added to the sections, which were incubated overnight at 4°C. Appropriate isotype matched rabbit or mouse antibodies at the same concentrations as the primary antibodies were used as isotype controls (R&D Systems, Inc., USA or Abcam Inc., USA). Next, the sections were incubated for 30 min at room temperature with the following secondary antibodies: Alexa 488-conjugated donkey anti-rabbit and Alexa 568-conjugated donkey anti-mouse IgG (Invitrogen, CA, USA). A drop of Prolong Gold antifade reagent with DAPI (Invitrogen, USA) was used to seal the coverslips. The patterns of immunoreactivity were visualized on a Zeiss LSM 710 type laser scanning confocal microscope (CARI ZEISS, Germany).

### Enzyme-linked Immunosorbent Assay (ELISA)

We quantified the secreted CCL20 protein levels in the conditioned human NP cell culture supernatants with a Human CCL20/MIP-3α Quantikine ELISA kit (R&D Systems, USA) according to the manufacturer’s protocol. All procedures were performed at room temperature, and the mean absorbances of the standards and samples were detected in duplicate. The minimum detectable dose (MDD) of the ELISA kit ranged from 0.10–0.87 pg/ml and the mean MDD was 0.47 pg/ml. The colorimetric reactions were analyzed at 450 nm on a Varioskan flash multifunction plate reader (Thermo scientific, Germany).

### Real-time PCR

The gene expression levels of CCL20 and CCR6 were analyzed by real-time fluorogenic RT-PCR on a Roche LightCycler2.0 (Germany). The PrimeScript® RT reagent Kit with gDNA Eraser (TaKaRa, Japan) was used for cDNA synthesis, and the CCL20 and CCR6 mRNA expression levels were calculated after normalization to the β-actin expression levels. The cDNA were detected with SYBR Green (TaKaRa, Japan) upon amplification with the following primers (TaKaRa, Japan):human CCL20, forward 5′-TTGATGTCAGTGCTGCTACTCCA-3′, and reverse 5′-TGTGTATCCAAGACAGCAGTCAAAG-3′;human CCR6, forward 5′-CAGTCAACAAGCCTGACCCTGTAA-3′,and reverse 5′-TCCTAATGGCCCACTACAACCTG-3′. We oatained the cycle threshold (Ct) data and used the 2^−△△Ct^ method to standardize the data to the β-actin expression levels.

### Flow Cytometry

Flow cytometry was used to evaluate the proportion of IL-17-producing cells in the PB samples from diagnosed degenerated IVD patients, as well as the surface expression of CCR6 on those cells. Briefly, heparinized peripheral whole blood (400 µl) was mixed with an equal volume of Roswell Park Memorial Institute 1640 medium and incubated for 4 h at 37°C, 5%CO_2_ in the presence of 25 ng/ml of phorbol myristate acetate (PMA) (LiankeBio, LK-CS0001), 1 µg/ml of ionomycin (LiankeBio, LK-CS0002), and 1.7 µg/ml of monensin (LiankeBio, LK-CS0004). PMA and ionomycin are pharmacological T cell-activating agents that mimic signals generated by the T-cell receptor (TCR) complex; these reagents are advantageous because they can stimulate T cells of any antigen specificity. Monensin was used to block intracellular transport mechanisms, thereby leading to an accumulation of cytokines in the cells. After the incubation, the cells were stained with APC-conjugated anti-human CCR6 (eBioscience, 17-1969-41), FITC-conjugated anti-human CD8 (eBioscience, 11-0086-41) and PE-Cy5.5-conjugated anti-human CD3 (eBioscience, 35-0036-41) monoclonal antibodies at room temperature for 15 min while shielded from light. The anti-human CD3 and CD8 monoclonal antibodies were used to delimit the CD4^+^ T cells because CD4 expression was diminished when the cells were activated by PMA. After surface staining, the cells were fixed and permeabilized (LiankeBio, LK-GAS003, FIX&PERM Kit) and stained with a PE-conjugated anti-human IL-17A monoclonal antibody (eBioscience, 12-7178-41). Isotype controls were used to enable correct compensation and confirm antibody specificity. The stained cells were analyzed by flow cytometry on a FACScan cytometer equipped with CellQuest software (BD Bioscience Pharmingen, San Jose, CA, USA). CD3^+^ T cell subsets were gated by flow cytometry, and the proportion of IL-17-producing cells (CD8^−^ (CD4^+^) IL-17^+^) was determined. We determined the rate of surface CCR6 expression upon cells in the CD8^−^ (CD4^+^) IL-17^+^ gate.

### Statistical Analysis

Continuous variables were expressed as the means ± SD. An unpaired student’s t test was used for the normally distributed variables. One-way ANOVA followed by a multiple-comparison test for subgroups by the least significance difference (LSD) was used to compare the mean among multiple groups. Pearson correlation analyses were applied to determine the dose- and time- dependencies. All statistical analyses were performed with SPSS 16.0 software for Windows (SPSS, Chicago, IL, USA). A two-tailed P-value<0.05 was set as the level of significance.

## Results

### Expression of CCL20 and TNF-α in the Degenerated IVD Tissues

**Figure 1:**
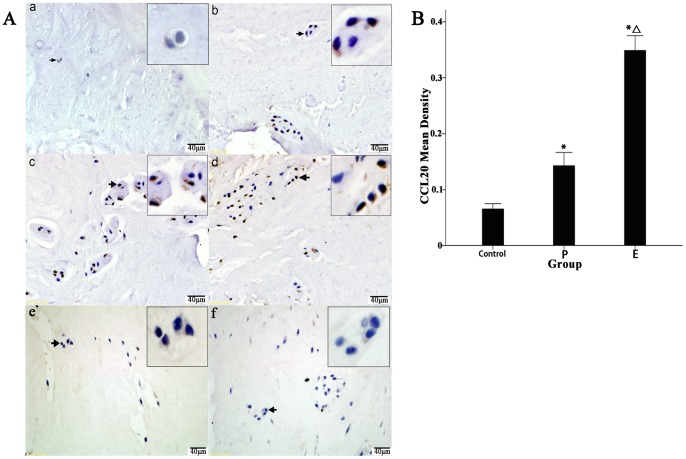
Immunohistochemical localization of CCL20 in human IVD tissues and comparison of mean density scores of the positive cells between the three groups. Fifty IVD tissue samples (Group P, n = 20 and Group E, n = 30) from disc degeneration patients and 3 from scoliosis patients were analyzed. Representative results from each group are shown. No distinct CCL20 expression was detected in the control (a). (b) and (c) represent the CCL20 expression analysis of the protrusion and extrusion groups, respectively. Inflammatory reactions on the edges of extruded IVD tissues are shown in (d). The magnifications of the positive cells were shown in the frame of the top right corner of (b), (c) and (d), respectively. (e) and (f) represent the isotype control of (b) and (c), respectively. Magnification, 400x; scale bars, 20 µm. Image-Pro Plus software was used to perform a quantitative analysis of the IHC results. Compared to the normal control, the expression levels of CCL20, shown as the mean densities of the positive cells, were significantly increased in the patient groups (B, *, P<0.01, significantly different from the control, *△, P<0.01, significantly different from Group E).

**Figure 2:**
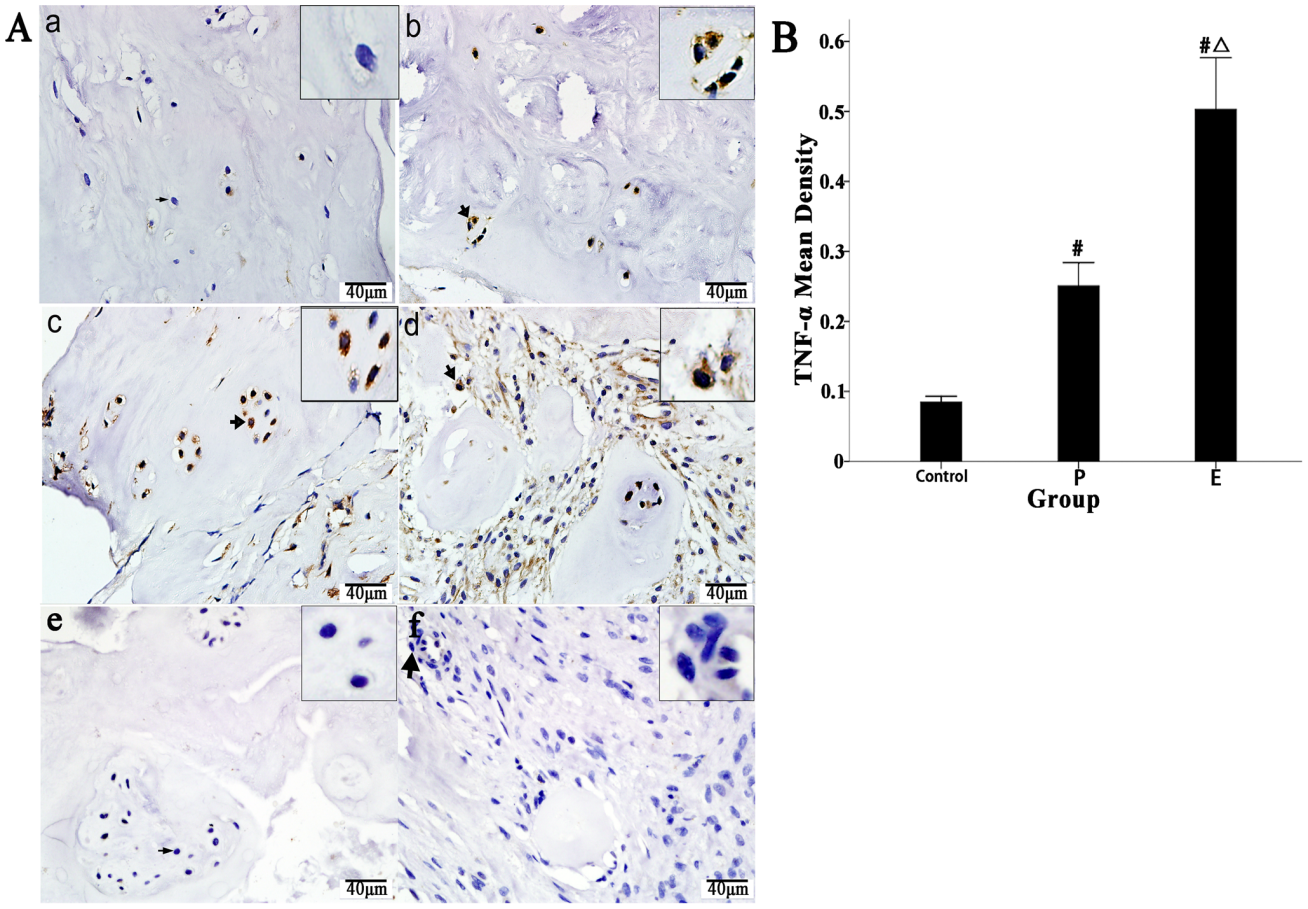
Immunohistochemical localization of TNF-α in human IVD tissues and comparison of mean density scores of the positive cells between the three groups. Fifty IVD tissue samples (Group P, n = 20 and Group E, n = 30) from disc degeneration patients and 3 from scoliosis patients were analyzed. Representative results from each group are shown. No distinct expression of TNF-α was detected in the control (a). (b) and (c) represent the TNF-α expression analysis of the protrusion and extrusion groups, respectively. (d) demonstrates many inflammatory cells on the edges of extruded IVD tissues that express TNF-α. The magnifications of the positive cells were shown in the frame of the top right corner of (b), (c) and (d), respectively. (e) and (f) represent the isotype control of (b) and (c), respectively. Magnification, 400x; scale bars, 20 µm. Image-Pro Plus software was used to perform a quantitative analysis of the IHC results. Compared to the normal control, the expression levels of TNF-α, shown as the mean densities of the positive cells, were significantly increased in the patient groups (B, #, P<0.01, significantly different from the control, #△, P<0.01, significantly different from Group E).

As shown in Figure 1A, CCL20 immunoreactivity was detected in the NP cells from degenerated IVD tissues. Different levels of CCL20 immunoreactivity were detected in the NP cells from Groups P and E. The degree of degeneration in Group P was conspicuously lower than in Group E, with fewer chondrocytes gathered in the cartilage cavities and no inflammatory reactions on the edges of the IVD tissues. In both Group P and Group E, there were many CCL20-producing NP cells, and there was no obvious difference in the numbers of CCL20-posotive cells between the groups (Figure 1A b and c). In the inflammatory regions of the Group E samples, there were many NP cells as well as CCL20-secreting inflammatory cells (Figure 1A d). Simultaneously, the NP cells from either group could also secret TNF-α, as did many inflammatory cells in the Group E samples (Figure 2A b, c and d); secretion of these cytokines was not detected in the normal control samples (Figure 1A a and Figure 2A a). Finally, we used Image-Pro Plus software to generate a quantitative analysis of the results. As shown in Figure 1B and 2B, when compared to the normal controls, the expression of CCL20 and TNF-α (shown as the mean densities of the positive cells and the mean density represents the average optical density of the object, which was defined as the expression intensity of those cytokines) in Groups P and E were significantly increased (CCL20: Group E, 0.34 vs. Group P, 0.14 vs. normal control, 0.06, p<0.001; TNF-α: Group E, 0.50 vs. Group P, 0.25 vs. normal control, 0.08, p<0.001). The results suggested that in the degenerated IVD tissues, the CCL20 expression is significantly up-regulated, which might attract more Th17 cells via CCL20/CCR6 axis. Additionally, the high expression levels of TNF-α and IL-17 could stimulate the NP cells to produce more CCL20, thus forming a positive chemotactic feedback loop.

### CD4-positive, IL-17A-positive and CCR6-positive Cells are Present in the Degenerated IVD Tissues

**Figure 3:**
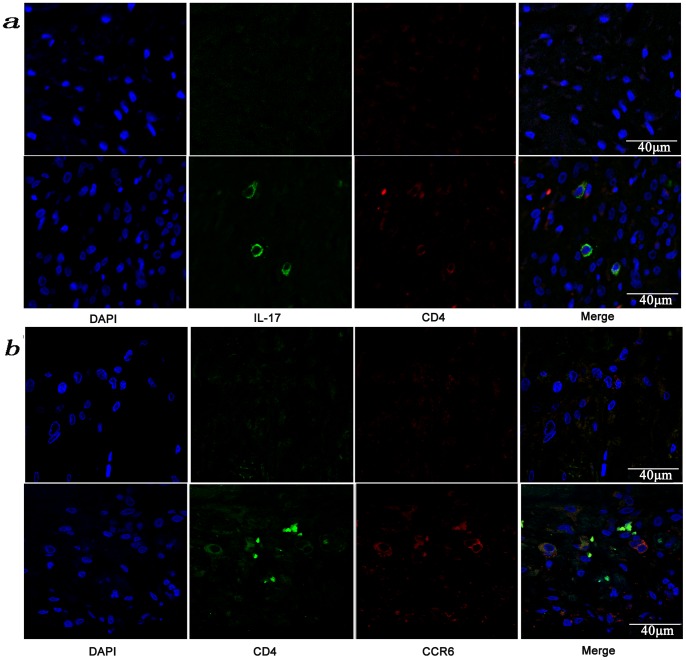
Presence of IL-17-producing cells in the degenerated IVD tissues. Fifty IVD tissue samples (Group P, n = 20 and Group E, n = 30) from disc degeneration patients and 3 from scoliosis patients were analyzed. In Group P or the control group, there were few or no positive cells (data not shown). The representative results of group E are shown. (a), IL-17-producing cells were detected by a rabbit anti-IL-17 polyclonal antibody and a mouse anti-CD4 monoclonal Ab, followed by secondary staining with an Alexa 488-conjugated donkey anti-rabbit and an Alexa 568-conjugated donkey anti-mouse IgG. DAPI mounting medium was used for nuclear staining. (b), surface CCR6 expression on the cells was detected with a rabbit anti CD4 monoclonal Ab and a mouse anti-CCR6 monoclonal Ab, followed by secondary staining with an Alexa 488-conjugated donkey anti-rabbit and an Alexa 568-conjugated donkey anti-mouse IgG. DAPI mounting medium was used for nuclear staining. In the top panel, green and red represents the expression of IL-17 and CD4, respectively, and the double-stained cells represent the IL-17-producing cells. In the bottom panel, green and red represents the expression of CD4 and CCR6, respectively, and the double-stained cells demonstrate the surface expression of CCR6 on T lymphocytes.

As shown in Figure 3, there was severe inflammation in the Group E IVD tissues as determined by the appearance of a large number of DAPI-stained cell nuclei. Some of the cells were both CD4^+^ and IL-17A^+^ and we considered those CD4^+^IL-17A^+^ cells to be IL-17-producing cells (Figure 3a). In contrast, there were few or no positive cells in the Group P and normal control samples. Then in the inflammatory reaction, the double-labeled immunofluorescent analysis of CD4 and CCR6 expression showed that some of the inflammatory cells were both CD4^+^ and CCR6^+^ (Figure 3b). As previous studies [18]–[20] showed that the chemokine receptor CCR6 was expressed on the surfaces of Th17 cells, we speculated that those CD4^+^CCR6^+^ cells might be IL-17-producing cells.

### Effects of IL-17A and TNF-α on CCL20 Secretion by NP Cells from Degenerated IVD

**Figure 4:**
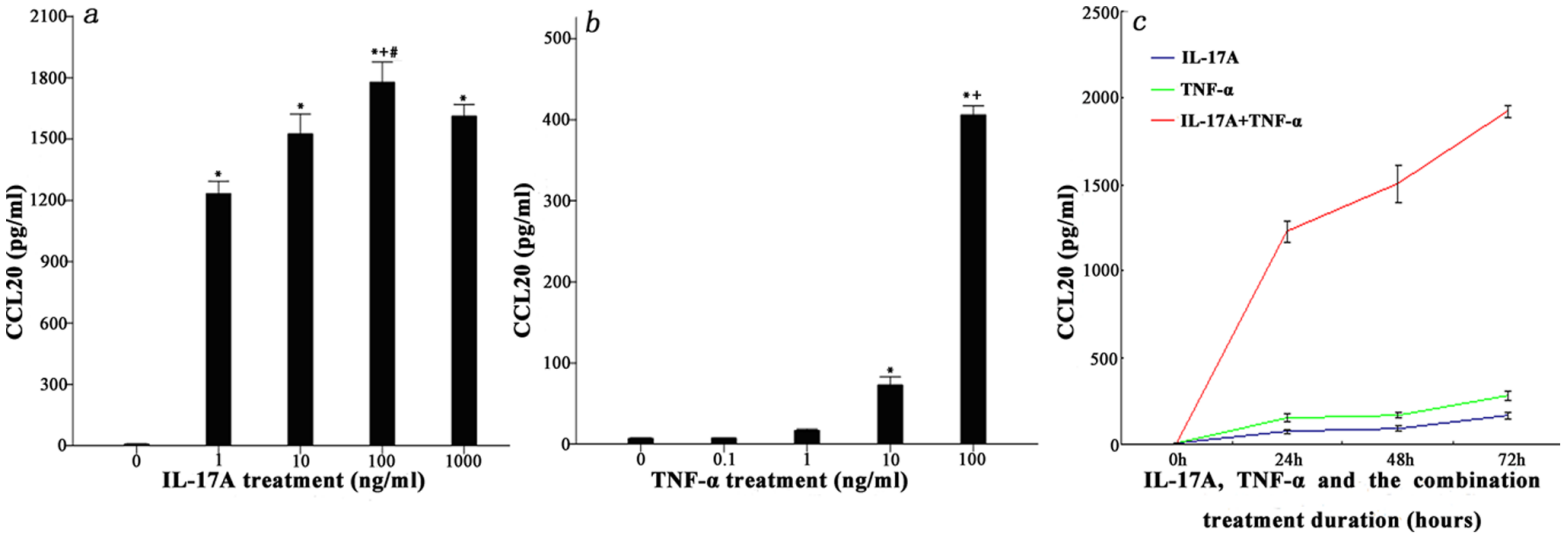
IL-17A, TNF-α, alone or in combination, can increase the secretion of CCL20 protein from NP cells in a dose- and time-dependent manner. Eight IVD tissue samples from disc degeneration patients were used as the primary cultures. NP cells were stimulated with the indicated concentrations of (a) IL-17A or (b) TNF-α for 48 hours. The cells were treated with the optimal concentrations of (c) IL-17A, TNF-α or both for 0, 24, 48 and 72 hours. The cell-free supernatants were harvested and the expression levels of CCL20 protein were quantified by ELISA. The data are representative of at least three separate experiments, performed in duplicate, and the mean protein levels and standard deviations (SD) are shown. Significant differences were detected by a least significance difference analysis (LSD) (P<0.05). There was statistically significant differences between each concentration of IL-17 when compared to the control; however, statistical significance was verified only for TNF-α concentrations higher than 10 ng/ml (IL-17 treatment: *, P<0.01, vs. control; +, P<0.001, vs. 10 ng/ml; ^#^, p<0.05, vs. 1000 ng/ml; TNF-α treatment: *, p<0.001, vs. control; +, p<0.001, vs. 10 ng/ml).

Both IL-17A and TNF-α cytokines can enhance CCL20 secretion in the NP cells from degenerated IVD tissues in a dose-dependent manner. IL-17A significantly enhanced CCL20 secretion by more than 100-fold over untreated cells, and the optimal concentration for IL-17A stimulation in the NP cell cultures was 100 ng/ml (Figure 4a). TNF-α stimulated a gradual increase in CCL20 secretion in a dose-dependent manner, and the optimal concentration for TNF-α stimulation was 100 ng/ml (Figure 4b). However the effect of TNF-α was not as pronounced as that of IL-17A. The optimal concentrations of IL-17A and TNF-α (100 ng/ml) were used independently to stimulate the NP cells for 24, 48 and 72 h; however, there were not highly obvious time-dependent effects on the production of CCL20. When the two cytokines were combined to stimulate the NP cells, the more significant increase in CCL20 production provided possible evidence for a synergistic correlation between IL-17A and TNF-α (Figure 4c).

### Effect of IL-17A, TNF-α on CCL20 mRNA Expression in Degenerated IVD NP Cells

**Figure 5:**
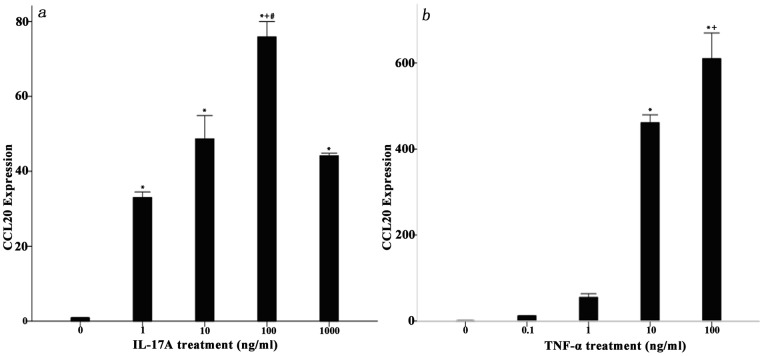
IL-17A and TNF-α increase CCL20 mRNA expression in NP cells in a dose-dependent manner. Eight IVD tissue samples from disc degeneration patients were used as the primary cultures. The cells were treated with the indicated concentrations of (a) IL-17A or (b)TNF-α for 48 hours. The expression levels of CCL20 mRNA were detected and the CCL20 transcripts were quantified by real-time RT-PCR analyses in all experiments. The data are representative of at least two separate experiments, performed in duplicate, and the mean increases in the mRNA expression were recorded, after which we evaluated the standard deviations (SD). Significant differences were detected by a least significance difference analysis (LSD) (P<0.05). The results correspond with the findings obtained in the ELISA tests. (IL-17 treatment: *, P<0.001, vs. control; +, P<0.05, vs. 10 ng/ml; ^#^, p<0.01, vs. 1000 ng/ml; TNF-α treatment: *, p<0.001, vs. control; +, p<0.01, vs. 10 ng/ml).

The NP cells that had been treated with IL-17A and TNF-α were used for the detection of CCL20 mRNA expression by RT-PCR. The data showed that the expression levels of CCL20 mRNA were dose-dependent (Figure 5) but not time-dependent (data was not shown). The data also showed that at a stimulating cytokine concentration of 100 ng/ml, the mRNA expression level was the highest, which concurred with the ELISA results. However, Kawaguchi [21] failed to detect the CCL20 mRNA expression in human herniated intervertebral discs. In the previous study, the mRNA level of CCL 20 was measured using RT-PCR method, but it was not found. The discrepant results between the study by Kawaguchi et al. and the current one may be attributed to the different experiment methods. In the previous study, the RNA was directly extracted from the degenerated IVD tissues, and RT-PCR was used to detect the expression of CCL20 mRNA. In the current study, we used IHC to detect the CCL20 expression in each samples and found that many NP cells were positively stained. Additionally, we cultured the NP cells and collected the supernatants to detect the CCL20 protein levels by ELISA. At the same time, RNA had been extracted from the NP cells to detect the CCL20 mRNA expression by RT-PCR. Our results demonstrated that the degenerated NP cells could secret abundant amounts of CCL20.

### Expression of CCR6 mRNA in PBMCs from Patients with Degenerated IVD and Healthy Controls

**Figure 6:**
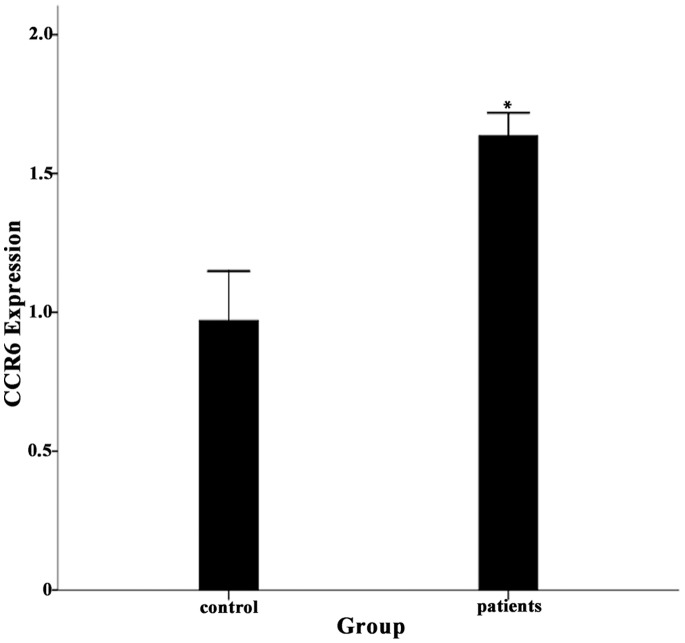
Expression of CCR6 mRNA in PBMCs from patients with degenerated IVD and and healthy controls. Fifty PB samples from disc degeneration patients and ten from healthy controls were used for PBMC isolation and detection of CCR6 mRNA expression. There was a significant difference in CCR6 mRNA expression levels between the isolated PBMCs from the patient group and those from the healthy control group. The CCR6 mRNA expression levels were quantified by real-time RT-PCR for all experiments. The data are representative of at least two separate experiments, performed in duplicate, and the average increase in the mRNA expression in the patient group was reported, after which the standard deviations (SD) was calculated. Significant differences were detected with the unpaired student’s t test (*, P<0.05, significantly different from the control).

We detected the expression levels of CCR6 mRNA in the isolated PBMCs from the patients with degenerated IVD and healthy controls. The results showed a significant difference in CCR6 mRNA levels between the patient group and the healthy control group (p<0.05, Figure 6). These data, combined with the flow cytometry analysis, suggested that the proportion of IL-17-producing cells and the expression of CCR6 were increased in the PB from patients with degenerated IVD, and that the PB might be a rich source of the IL-17-producing cells that traffic to the IVD lesions via the CCL20/CCR6 system.

### Expression of Elevated Th17 Cells and CCR6 in Peripheral Blood Samples of patients with Degenerated IVD

**Figure 7:**
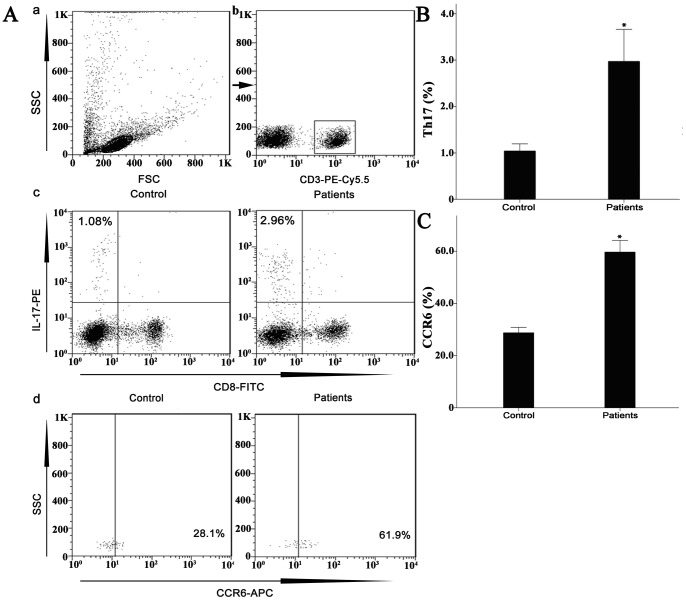
Circulating percentages of Th17 cells and CCR6-positive cells in peripheral blood are increased in IVD degenerated patients when compared with controls. Heparinized peripheral whole blood cells from 20 patients and 15 healthy controls were stimulated with phorbol myristate acetate (PMA), ionomycin, and monensin for 4 h and subsequently stained with fluorochrome-labeled antibodies as described in Materials and Methods. A(a) Lymphocytes were gated by flow cytometry. A(b) CD3^+^ T subsets were gated by flow cytometry; the plots in the inset box represent the CD3^+^ T cells. A(c) Representative IL-17 expression levels in the CD3^+^CD8^−^T subsets (CD4^+^ T subsets) from each group are shown. The percentages of positive cells are shown in the upper left panels. A(d) Representative surface CCR6 expression levels on the CD3^+^CD8^−^IL-17^+^ subsets from each group are shown. The percentages of positive cells are shown in the right panel. (B) The percentage of circulating Th17 cells was significantly higher in IVD degenerated patients (2.973±0.689%) than in the control group (1.039±0.156%; *, p<0.001, significantly different from the control). (C) The rate of surface CCR6 expression on circulating Th17 cells was significantly higher in patients with degenerated IVD (59.69±4.48%) than the control group (28.75±2.09%; *, p<0.001, significantly different from the control).

We analyzed the frequencies of peripheral Th17 cells based on the cytokine expression patterns after a short-term *in vitro* stimulation by PMA and ionomycin. Lymphocytes (Figure 7A a) and CD3^+^ T cell subsets (Figure 7A b) were gated by flow cytometry, and the proportions of IL-17-producing cells (CD8^−^IL-17^+^) were determined from the subsets of CD3^+^ cells (Figure 7A c). Afterwards, we determined the rate of surface CCR6 expression on the CD8^−^IL-17^+^ gated cells, (Figure 7A d). A typical dot plot of circulating IL-17-producing (CD3^+^CD8^−^IL-17^+^) cells and the rates of surface CCR6 expression in cells from representative degenerated IVD patients and healthy controls are shown in Figure 7A c and 7A d, respectively. The statistical analysis of the flow cytometry data is shown in Figure 7B and 7C. Compared to the healthy controls, the percentage of peripheral IL-17-producing cells significantly increased in the patients with degenerated IVD (1.039±0.156% vs. 2.973±0.689%, P<0.0001). The rate of surface CCR6 expression on the IL-17-producing cells (CD4^+^IL-17^+^) was profoundly increased in the patients with degenerated IVD when compared to the healthy controls (28.75±2.09% vs. 59.69±4.48%, P<0.0001). The results indicate that in the peripheral blood from patients with degenerated IVD, the frequency of IL-17-producing cells is significantly increased and these cells express high levels of surface CCR6, the specific ligand of CCL20. These cells could be a rich source of the IL-17-producing cells that are present in the local NP tissues.

## Discussion

In the current study, we confirmed with IHC and an NP cell monolayer culture system that NP cells can produce abundant amounts of CCL20 (a specific ligand for CCR6). Additionally, we concluded that the Th17 associated cytokines (IL-17A and TNF-α) dramatically increased the production of CCL20 when added to the NP cell culture media alone or in combination. Another interesting observation was of serious inflammation on the edges of the herniated disc tissues; there was very little inflammation on the extruded disc tissues and none in the control tissues that were obtained from scoliosis patients. Furthermore, we confirmed a high frequency of CD4^+^, IL-17A^+^, CCR6^+^ cells in the herniated disc tissues, especially in the inflamed portion, but few or no CD4^+^, IL-17A^+^, CCR6^+^ cells in the extruded disc tissues. We determined that the CD4^+^, IL-17A^+^, CCR6^+^ cells were highly likely to be IL-17-producing cells, because we employed a double immunofluorescence staining procedure and discovered that many of the cells were CD4^+^IL^−^17A^+^ or CD4^+^CCR6^+^. Next, we performed a RT-PCR analysis to determine the CCL20 mRNA levels in the cultured NP cells and the CCR6 levels in the PBMCs, and we came to know that the results were in accordance with the above-mentioned findings. Finally, a flow cytometric analysis showed that the frequency of Th17 cells and the rate of surface CCR6 expression on those cells were higher in the PB from patients than from the healthy controls.

These results provide us a better understanding of the Th17 cells’ roles in the inflammatory process in degenerated discs, as well as a possible mechanism of Th17 cell trafficking to the lesion area. First, in the PB from the degenerated disc patients, the increased expression levels of CCR6 protein and mRNA are in accordance with previous studies, as a chemokine receptor and a marker to identify Th17 cells [19], [20]. Second, most of the NP cells from the extruded and herniated patient group could produce abundant amounts of CCL20. Accordingly, we suggest that the CCL20 expressed by the NP cells could attract Th17 cells from the peripheral blood. Indeed, we found many CD4^+^IL-17A^+^, CD4^+^CCR6^+^ cells in the lesions of the degenerated discs, and a study by Pène [18] also suggested that CD4^+^CCR6^+^ might be a characteristic marker of IL-17-producing cells. Furthermore, this discovery is supported by the studies of Shamji [22] and Gabr [23], which suggest that IL-17A plays an important role in the inflammatory reactions in the degenerated discs by affecting the production of many inflammatory mediators; however, these studies did not provide strong evidence that IL-17A was secreted by the Th17 cells or a possible mechanism for the trafficking of Th17 cells to the degenerated disc. Thus, our original experimental design intended to provide the necessary evidence and to explore the possible mechanisms. Certainly, there were some limitations in our experimental design. First, we did not purify Th17 cells from the degenerated discs and chose to use a double-labeled immunofluorescence technique to ensure the detection of Th17 cells in the locally degenerated discs; second, we did not perform a chemotaxis experiment to ensure that the degenerated CCL20-secreting NP cells could attract the CCR6-expressing Th17 cells either in *vivo* or in *vitro*. Thus, in future studies we should focus on seeking the appropriate methods for Th17 cell purification and direct chemotaxis experiments to confirm the suggested mechanism.

In previous studies, histological studies of degenerated discs demonstrated a dense inflammatory cellular infiltration comprised mainly of macrophages, which are a source of many of the previously mentioned cytokines [28]–[31]. Similarly, noticeable elevations in the levels pro-inflammatory cytokines, including nitric oxide (NOx), prostaglandin E2 (PGE2), interleukin-6 (IL-6) and interleukin-8 (IL-8) [32] and cytokines that are important to the regulation of pathologies, including IFN-γ, TNF-α and IL-1β [33]–[36] have been documented in the lesions of extruded and herniated discs. Of these cytokines, TNF-α, a potent regulator of angiogenesis [37], can up-regulate the expression of vascular endothelial growth factor (VEGF), which results in the formation of new blood vessels [38], [39]. Indeed, in our study we found that the herniated discs were surrounded by granular tissues that were characterized by newly formed vessels and inflammatory cell infiltrates, as reported in previous studies [40], [41].

Significantly, the Th17 cells, as previously mentioned, are capable of producing abundant pro-inflammatory cytokines, including IL-17A, TNF-α, IL-22 and IL-26. Furthermore, other studies have proven that the addition of Th17 associated cytokines to NP cell culture medium upregulate the production of NOx, PEG2 and IL-6 [23]. Of further interest, during our experiments with NP cell monolayer cultures, the addition of Th17 associated cytokines (IL-17A and TNF-α) to the culture medium resulted in dramatically increased CCL20 production. Currently, we know that CCL20 is also known as the macrophage inflammatory protein 3α (MIP-3α); it is a 70-amino-acid chemokine that binds exclusively to the chemokine receptor 6, which is specifically expressed on the surface of Th17 cells (18–20). CCL20 has been reported in many immune mediated inflammatory diseases, such as rheumatoid arthritis [10], [11], psoriasis [12], [13], asthma [14], [15] and inflammatory bowel disease [16], [17]. In the present study, we suggest that CCL20 was expressed after IVD degeneration, because we found that the herniated discs were surrounded by varying degrees of inflammatory cells and newly formed blood vessels that were not obvious in the extruded discs. However, the expression of CCL20 did not appear to have a strong influence. Thus, we speculated that signal pathway was activated in the NP cells to induce the production of CCL20, and that there was less accumulation of CCL20 before the rupture of the annulus fibrosus. Once the annulus fibrosus ruptured, abundant amounts of CCL20 were released that attracted CCR6-expressing Th17 cells from the peripheral blood. After Th17 cells were transferred via chemotaxis to the lesion areas, they could secrete many inflammatory cytokines, leading to the appearance of a cytokine milieu with the cytokines produced by macrophages or other inflammatory cells. This cytokine milieu promotes the inflammatory process and stimulates the production of CCL20, including the trafficking of more Th17 cells to the inflammatory lesion. As a result, we have a potential explanation for how the CCR6-positive Th17 cells maintain a continuous presence in the degenerated discs through a positive chemotactic feedback loop.

Previous reports have stated that, in a mouse model of rheumatoid arthritis, the addition of anti-CCR6 monoclonal antibodies inhibited the trafficking of Th17 cells to the arthritic joint and impeded early disease development [10]. This underscores the point that through the blocking of CCR6 on Th17 cells during the initial process of IVD degeneration or reductions in CCL20 production by modifying the cytokine milieu in the degenerated discs, we could prevent Th17 cell trafficking via the CCL20/CCR6 chemotactic pathway, which might be useful to cure or prevent the immune induced inflammation. Another report showed that when specific inhibitors of p38-MAPK, p42/44-MAPK, or SAPK/JNK were added to cultured endometrial stromal cells, CCL20 production was dramatically diminished [42]. Although this is not directly related to our research, we assume that a deeper understanding about the mechanism involved would open gateways for future findings.

### Conclusions

In conclusion, we found that the NP cells could produce abundant amounts of CCL20, and that Th17 associated cytokines (IL-17A and TNF-α) can up-regulate CCL20 production. In the pathological lesions of the degenerated discs, we observed increased frequencies of IL-17-producing cells (CD4^+^IL-17A^+^ or CD4^+^CCR6^+^) and CCR6 mRNA expression levels in the PBMCs. Thus, as a possible mechanism, we suggest that Th17 cells trafficked to the lesions via CCL20/CCR6 pathway. Further studies are required to seek appropriate methods for Th17 cell purifications and to perform chemotaxis experiments directly or by using animal models to confirm this mechanism.
